# Kindness Isn’t Just about Being Nice: The Value Proposition of Kindness as Viewed through the Lens of Incivility in the Healthcare Workplace

**DOI:** 10.3390/bs13060457

**Published:** 2023-06-01

**Authors:** David A. Fryburg

**Affiliations:** Envision Kindness, East Lyme, CT 06333, USA; david@envisionkindness.org

**Keywords:** stress, incivility, disruptive behavior, healthcare, medical error, kindness, connection, patient, provider, workplace

## Abstract

The healthcare workplace is a high-stress environment. All stakeholders, including patients and providers, display evidence of that stress. High stress has several effects. Even acutely, stress can negatively affect cognitive function, worsening diagnostic acumen, decision-making, and problem-solving. It decreases helpfulness. As stress increases, it can progress to burnout and more severe mental health consequences, including depression and suicide. One of the consequences (and causes) of stress is incivility. Both patients and staff can manifest these unkind behaviors, which in turn have been shown to cause medical errors. The human cost of errors is enormous, reflected in thousands of lives impacted every year. The economic cost is also enormous, costing at least several billion dollars annually. The warrant for promoting kindness, therefore, is enormous. Kindness creates positive interpersonal connections, which, in turn, buffers stress and fosters resilience. Kindness, therefore, is not just a nice thing to do: it is critically important in the workplace. Ways to promote kindness, including leadership modeling positive behaviors as well as the deterrence of negative behaviors, are essential. A new approach using kindness media is described. It uplifts patients and staff, decreases irritation and stress, and increases happiness, calmness, and feeling connected to others.

## 1. Introduction

This article is about the effects of stress, and, in particular, how people treat each other, on performance in the workplace. The focus is a healthcare workplace in which optimal performance is critical to maximize positive outcomes for patients as well as to maintain staff morale and meaning from work. This paper reflects the assembly of a broad literature base to show how stress, and specifically, incivility, affects cognitive function and team dynamics. In turn, incivility and other disruptive behaviors depress quality decision-making and markedly increase errors. 

Beyond providing a general readership with an integrated review of a largely avoidable circumstance leading to medical errors, the major goal of this article is, as a position paper, to reach three specific groups of stakeholders. These include: 1. decision-makers in health care facilities; 2. those who administer insurance payments for care or malpractice; and 3. those who lead businesses that pay for these services on behalf of their employees.

The rationale for this position paper is to prompt thinking about how a shift in the way people treat each other can have significant and very practical ramifications in one of the most important workplace settings. *It is to provide a warrant to take action.* Taking this to a personal level, readers need to ask themselves two questions. First, would I (the reader) want to have a doctor or nurse or any other provider of care have lower than peak performance when these professionals are seeing me or my parents, children, friends, or anyone else close to me? (and, of course, co-workers, strangers, et al.). Second, beyond the human consequences, does the actual cost of incivility in health care drive investing in kindness to create a lower-stress, higher-performance culture? What is the value proposition of that investment?

Although there are many stressors in healthcare, by understanding incivility in the healthcare workplace, the case to be made for promoting prosocial behaviors—for kindness—is substantial. The human and economic value proposition of kindness is estimated.

## 2. Scope of Medical Errors as a Problem: Taking Good Care of the Customer (Patient)

To begin assembling the case, it is important first to understand the scope of medical errors and the role of stress in creating those errors. A seminal report issued at the turn of this century by the Institute of Medicine entitled “To Err Is Human” presented the healthcare profession with a shocking statistic: medical errors were a very common cause of death. At the time, it was estimated that between 44,000 and 98,000 people died due to medical errors annually, exceeding automobile accidents, breast cancer, and AIDS [1]. Subsequent estimates have placed the magnitude of this problem even higher, up to >400,000 deaths per year in the United States [2].

Beyond death, there is a gamut of other non-fatal and serious outcomes from errors that may be 10–20 fold that of fatal errors [2]. For example, more than 12 million patients annually experience a diagnostic error, about half of which have the potential to cause harm [3]. Or that ~9% of hospitalizations have an adverse event such as an untoward reaction to a drug or hospital-acquired infection [4]. Medication errors are particularly common and account for approximately 700,000 emergency room visits and 120,000 hospital admissions [5], a rate that has been sustained, if not increased [6].

In Massachusetts, USA, the Betsy Lehman Center For Patient Safety quantified that there were almost 62,000 preventable harm events in just one year alone, at the cost of over USD 600 million [7]. Given that the population of Massachusetts is ~7 million people across the US, that would translate to approximately 3 million preventable harm events each year at the cost of USD 30 billion. This independent estimate is in accord with a projection from the National Quality Forum in 2010 [8] and Adler and colleagues [9]. The psychological burden of these errors could be steep, depending, in large part, on how much communication there was between patient and provider about the error [10].

This problem is observed in many countries. A recent report from the OECD estimates that the annual total cost for preventable harm in developed countries is approximately USD 600 billion or approximately 10% of health care expenditures [11]; for the US, this would equate to ~USD 300 billion. These direct costs are passed onto the insurers, whether private or governmental. These estimates do not include societal costs, i.e., disability, lost work, etc. That amounts to much more.

Communication problems are a major factor contributing to medical errors [12]; it has been estimated that 70% of errors are caused by them [13]. In addition, because health care requires a team approach, effective communication is the cornerstone of high-quality teamwork and, therefore, care [14].

## 3. Stress and Burnout in Health Care

To better understand how these errors occur, it is important to understand the impact of stress. Clearly, providers of health care are burdened with a lot of stress. As an occupational hazard, doctors, nurses, and many other providers are tasked with making decisions about someone else’s health while simultaneously being burdened in a high-demand-low agency setting. Providers must also manage the highly contagious emotions of their patients, many of whom port their stressors into an office visit [15,16].

Survey studies reveal that, in addition to their own personal stressors, patients have anxiety regarding whether the provider is going to care about them as well as find something wrong with them. We see this anxiety manifest physiologically in “white coat hypertension”, which occurs in at least 15–30% of patients [17].

In addition to needing to maintain a flow of patients so that they can satisfy patients and generate revenue, providers need to document the visit in electronic health records and perhaps negotiate with insurance providers [18]. Thus a 15 min encounter often has much more in the way of required administrative tasks that collectively lead to 50 to 60 h weeks. In addition to cross-cultural survey reports attesting to how providers feel stressed [19], another indicator of the pressure they are experiencing is the median time to interrupt a patient when the patient has an agenda: 11 s [20].

Kushnir and colleagues observed that family physicians who are stressed or in a bad mood listen (and talk) less with patients, write more prescriptions, and refer more often than those with positive affect [21]. That is, physicians in more negative moods spent more money and took more patient and other physician time, which is consistent with the literature on the opposite effects of patient-centered care [22,23,24]. The authors concluded that the more time spent on quality communication, the better the outcomes as well as the economics.

For providers, these chronic stressors have serious consequences. First, when prolonged, they promote burnout, depression, and suicide at rates double that of the general population [25,26,27]. Burned-out providers are more likely to have lower productivity because of absenteeism [28] and eventually leave the profession, which in turn generates staff shortages and adds more stress to the remaining staff. Burnout was epidemic in health care even before the COVID pandemic, the prevalence was 40–50% [29,30,31] and is associated with the intention to retire early [32,33]. The pre-pandemic annual cost of burnout in the USA has been estimated at almost USD 5 billion [34], which is largely reflective of lost productivity and replacing staff.

Second, caregivers who are burned out are much more likely to make errors and risk harming the patient [28,29,30,35,36,37]. Burnout has been mostly assessed by the Maslach Burnout Inventory (MBI), which has three major categories: emotional exhaustion, depersonalization, and personal accomplishment. Shanafelt and colleagues found that MBI-defined burned-out surgeons had double the risk of committing a major medical error in the previous three months than those who were not burned out [29]. It is important to note that in this study, major medical errors were reported voluntarily, which likely reflects underreporting of the true risk. Similar conclusions were made for those suffering from depersonalization.

As for physicians, the same has been observed in nurses [28,35,38] and across different medical specialties [36]. Moreover, the effect of burnout on medical errors transcends nationality and, therefore, healthcare systems—it is observed in many different countries [19,39] and has been shown to impact patient safety in meta-analyses [40,41].

It is particularly noteworthy that the surgeons who recognized their error most commonly attributed it to “a lapse in judgment” far more often than system-related issues [29,36].

## 4. Disruptive Behaviors in the Health Care Workplace

Incivility and related undesirable (and unkind) behaviors are a natural outcome of stress as well as a cause of stress. High-quality care requires strong, positive relationships among team members. Communication is key—taking the time to articulate information clearly coupled with active and empathetic listening to others is absolutely essential. The simplest team, of course, is the provider and the patient. They form a partnership in which quality communication is mandatory. Ultimately, they need to trust one another [42]; the cornerstone of trust, particularly for patients to caregivers, is that they listen. It is the tangible mark of caring.

In more complex teams that require multiple expertise (such as surgical teams comprised of physicians, nurses, aids, pharmacists, therapists, etc.), quality communication becomes even more essential [43]. That is, to minimize hand-off errors, have a shared understanding of the care plan, and coordinate its execution, effective, consistent, and compassionate communication is required. As with the simpler relationship between a patient and provider, a central component of multi-disciplinary teams is that team members need to trust one another [14,44].

Yet, in a stressful environment, quality communication can suffer. Both the person speaking, as well as the listener can be affected [45]. As a substantial portion of communication is non-verbal [12,46], and stress can affect both the content as well as the tone and prosody of speech and facial expressions [45,47], it is critical to recognize how important this “soft” aspect of interaction can be in the health care workplace. In addition, as even mild stressors affect cognitive processing (see below), it is easy to see how stress will disrupt the quality of communication and, therefore, care.

Beyond heavy workloads, restricted agency, and the responsibility for someone else’s health, another major class of stressors in health care is disruptive behavior. Disruptive behaviors include more blatant and active types, such as bullying, physical violence, discrimination, harassment, anger [33,48], as well as incivility, which is less directed.

In a seminal paper on the subject of workplace incivility, Andersson and Pearson define incivility as “low-intensity deviant behavior with ambiguous intent to harm the target, in violation of workplace norms for mutual respect. Uncivil behaviors are characteristically rude and discourteous, displaying a lack of regard for others” [49]. With regard to healthcare, there are two important facets of incivility to consider: between and among staff members and between the patient and provider.

It has been known for quite some time that incivility and other disruptive behaviors were commonplace in health care. They occur between patients (and their families) and staff, as well as between and among staff [50]. They are too often experienced by medical and nursing students and those in training after graduation [51,52].

Multiple studies have documented disruptive behaviors and examined their relevance to outcomes. Lucian Leape described the process as follows: “*Quality suffers when caregivers do not work in teams. Disrespect saps meaning and satisfaction from daily work and is one reason nurses experience burnout, resign from hospitals, or leave nursing altogether. Lack of respect poisons the well of collegiality and cooperation, undermines morale, and inhibits transparency and feedback. It is a major barrier to health care organizations becoming collaborative, integrated, supportive centers of patient-centered care*” [53].

Rosenstein and O’Daniel [13] surveyed hospitals in the western US of Voluntary Hospitals of America (VHA). A total of 4530 staff responded, approximately a 3:1 ratio of nurses to physicians. In that early study, the vast majority of respondents reported having ever witnessed disruptive behavior by physicians (77%) as well as by nurses (65%). The respondents also clearly linked the disruptive behaviors to negative outcomes for staff, such as stress (94%), reduced team collaboration (89%), reduced communication (91%), and impaired RNs and MDs (99%). A significant majority (67%) thought that there was a link between these behaviors and significant adverse events, which included death. Finally, 18% were aware of a specific adverse event that occurred because of disruptive behavior.

Westbrook and colleagues surveyed staff in seven tertiary care metropolitan hospitals in Australia using the Negative Acts Questionnaire [54] focused on disruptive behaviors among staff [55]. A total of 5178 staff responded (out of an invited 15,213). Of these, over 4600 (>89%) reported experiencing incivility of bullying within the past year; in 2009, staff members (39%) who reported experiencing disruptive behaviors at least weekly, a comparable number also reported that it negatively affected their well-being to a moderate or major extent.

Teamwork also suffered—55% responded that it had a moderate or major negative effect. Similarly, almost 50% of respondents stated that disruptive behavior decreased the quality of care to a moderate or major degree. Respondents who also reported (having) “speaking up skills” experienced a less deleterious effect on both teamwork and quality of care.

Lim and colleagues adopted Rosenstein and O’Daniel’s survey instrument to assess the incidence of disruptive behavior in Singaporean health care [56]. A total of 500 nurses and physicians responded, yielding an ~40% response rate. Approximately 95% of respondents had witnessed at least one form of disruptive behavior, which included rudeness, condescending remarks, facial expressions, outbursts (yelling), harassment, throwing objects, etc. Of the respondents, 34% witnessed these behaviors on a weekly or more frequent basis. These behaviors were reported to commonly result in negative employee outcomes as well as negative patient outcomes. Similar results have been observed by Oppel and colleagues in the US [57].

Cooper and colleagues examined how patient complaints about surgeons’ disruptive behaviors would predict later errors [58]. In this study, they examined how two years of prior complaints related to postoperative surgical and medical complications within 30 days after the surgery. They reported that surgeons with a greater number of complaints had a significantly increased risk of later errors. Separated into quartiles of complaints, the authors estimated that if the surgeon were in the three highest quartiles of patient complaints, compared to those in the lowest quartile, then 426 complications of surgery would have been avoided. A similar observation has also been made by Lagoo and colleagues [59].

These studies have largely focused on incivility that was displayed by a provider staff member. Yet disruptive behaviors from patients, families, and other visitors are another major source of stress for staff. In a large survey undertaken in a single academic medical center, ~23% of physicians reported experiencing “mistreatment” (including verbal abuse, physical violence or threats of violence, or sexual harassment) from patients and/or family members during the previous year, a finding that was linked to increased risk of burnout and intention to leave the profession [60,61,62]. Women and members of minorities, in general, are at greater risk of experiencing mistreatment.

Hatfield and colleagues recently documented that, at an academic medical center, verbal threats from patients or families were reported by 70% of healthcare practitioners within the previous year, with threats of physical harm or actual physical harm relayed by 23% and 12% of HCPs [63]. In another survey study at a large academic medical center, Meese and colleagues reported that approximately 10% of all clinical staff reported patient mistreatment as a major stressor, which was experienced most by nurses (19%). This problem is not isolated—in a meta-analysis that encompassed reports from 30 countries, it was found that ~20% of staff experienced workplace violence on an annual basis [64]. As expected, with a reported increase in errors, incivility decreases organizational efficiency in the healthcare workplace [61].

It should be emphasized that the problem of incivility is societal. It is considered a major problem by most Americans that is increasingly becoming worse [65]. It is not an American phenomenon—it is seen in the UK and elsewhere [66]. Incivility (disruptive behaviors) often begets retaliatory behavior by the recipient [67,68]. Negative behaviors spread, creating more stress and perpetuating diminished teamwork, communication, cognitive function, and work satisfaction.

## 5. Experimental Effects of Incivility

Although there is consensus among thousands of observers that disruptive behaviors are associated with, and likely causal in, creating medical errors and negative outcomes for patients, these are complex scenarios. That is, is it stress that causes incivility and that burned-out clinicians, who are more prone to commit errors and are also more likely to exhibit disruptive behaviors? Or does incivility directly cause errors?

A set of experimental studies have attempted to address the immediate impact of disruptive behaviors. Katz and colleagues used the setting of simulated surgeries to examine the effects of witnessed rudeness on the performance of anesthesiology residents [69]. In this study, residents are separated into two groups. In one group, the surgeon enters the simulated operating room and is polite and professional; in the other group, the surgeon is rude to the nurses but not to the anesthesiology residents.

The surgery proceeds, and the simulated patient has a drop in blood pressure due to an intra-abdominal hemorrhage. The residents are then judged by how they respond. Overall, 91% of the polite/professional surgeon pass, while only 64% of the residents exposed to the rude surgeon pass, largely due to not recognizing the problem (diagnosing it) and reacting appropriately. The reasons that they fail more often include less frequent: decrease in anesthetic, insertion of another IV, and ordering blood for transfusion. In addition, they showed much less interest in communicating with the surgeon. When asked after the simulation re: their own performance, the residents in the rude surgeon group thought that they performed well [69].

Riskin and colleagues performed a similar study in a neonatal ICU (NICU) simulation that involved both nurses and pediatricians [70]. In this case, a parent-actor entered the simulation and, in the rude experimental group and stated: “I knew we should have gone to a better hospital in which they do not practice third-world medicine”. The simulation proceeds—and those exposed to the parent-actor had significant declines in diagnostic planning, therapy score, teamwork, communication, etc. The effect appeared to last at least until the end of the day.

Cooper and colleagues conducted a series of studies that examined the effects of witnessed rudeness on diagnostic abilities [58]. In this series, witnessing disruptive behavior induced negative arousal and caused trainees to anchor or become stuck for a longer period of time on a specific (and wrong) diagnosis. The narrower perspective-taking yielded a delay in establishing the correct diagnosis and course of action.

Woolum and colleagues studied dental students who witnessed a lab manager in an unrelated and rude or pleasant interaction prior to a surgical task [71]. They found that those exposed to this rude interaction were cognitively depleted, performed significantly worse than those in the control condition, and made significantly more errors. Those who were not cognitively depleted did not exhibit the same augmentation in error rates.

Finally, Mamede and colleagues (BMJ 2016) studied the effect of described (not experienced) patient disruptive behavior on the resident’s ability to attain the correct diagnosis [72]. In clinical vignettes of either difficult or neutral patients, residents had a significantly lower diagnostic score for difficult patients than neutral patients.

Collectively, these experimental interventions give significant credence to the observation of many medical professionals: disruptive behaviors are causal in creating medical errors. It is important to emphasize that the person affected is NOT limited to the recipient or target of these behaviors. Simply being exposed to them, as many other staff would, would likely decrease the quality of care rendered, and that increases the effect manifold in which multiple team members have impaired function.

## 6. Effect of Rudeness on Cognitive Function

It has been known for a long time how incivility affects performance in non-medical fields. Porath and colleagues have described how rudeness hijacks cognitive resources. Beyond any volitional or recognized choice, rudeness decreases working memory and attention and stifles creativity and helpfulness [73]. Working memory, in particular, is involved in problem-solving and decision-making and is specifically impaired [73].

An illustrative experiment relevant to medicine is the “invisible gorilla” study. In that study, participants are asked to count the number of passes of a basketball. While doing that, a person in a gorilla suit passes across the field of the screen. In a group that had been exposed to incivility, they recognized the gorilla only 20% as often as the control group [74]. Using the invisible gorilla approach, Drew and colleagues examined how frequently experienced radiologists missed the gorilla on a CT scan: 83% of the time [75]. Given that radiologists have a high degree of burnout [76], it is very likely that many have inattentional blindness.

One explanation is that after exposure to incivility, the person spends cognitive resources continuing to think about the experience [72,77]. Alternatively, or perhaps in addition, incivility could be looked at as a potent stressor in humans. As stressors, in general, negatively affect cognitive function in animals, and other stressors in humans impair cognitive function [78,79,80,81,82], incivility likely impedes cognitive function subconsciously as part of a general stress response.

## 7. The Vicious Cycle of Stress and Disruptive Behavior in Health Care

**Figure 1:**
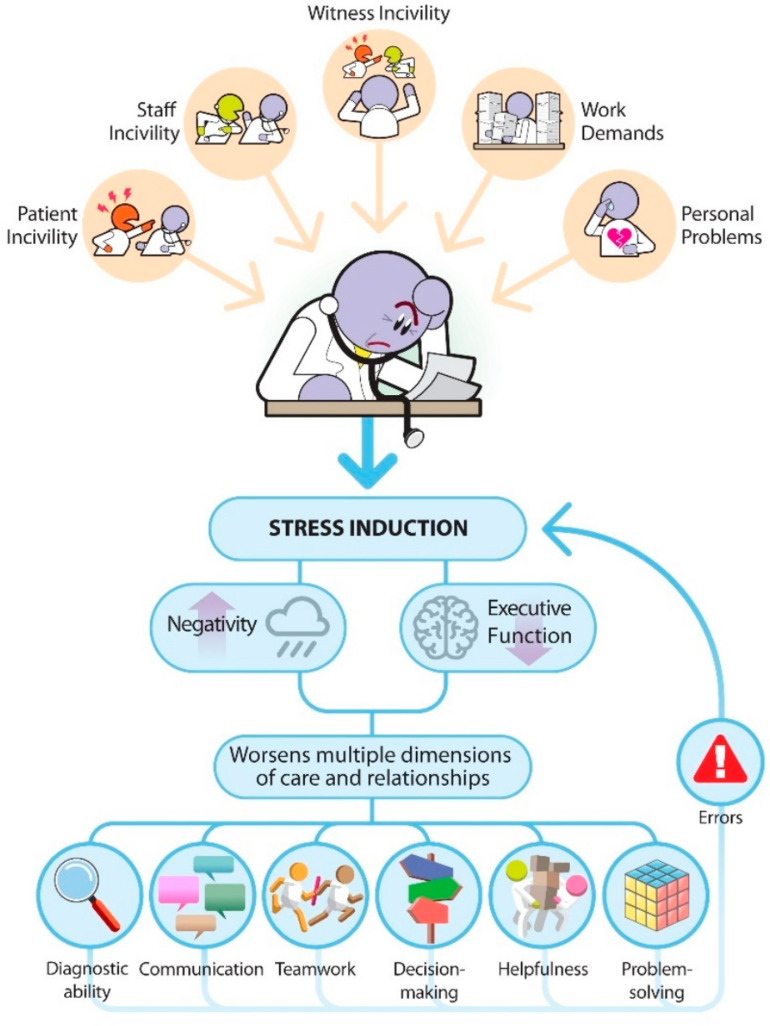
An integrative literature view as how incivility, along with other stressors, leads to medical errors. In the figure, the depicted healthcare provider is burdened with a differential mixture of stressors. In addition to their own personal issues and work demands, they must be able to manage disruptive behaviors in the workplace. These can be directly received from patients or other staff members or simply witnessed. As stress is induced, the negative effect increases, and cognitive function decreases. These, in turn, affect a variety of critical functions necessary to provide good care, including diagnostic ability, communication, teamwork, etc. As quality decreases and errors are committed, additional stress is induced. Depending on the severity of the error, the clinician can become a second victim, adding more stress. If sustained, burnout, depression, and other severe consequences can ensue. This is a vicious cycle.

Figure 1 integrates much of the aforementioned information. We imagine our prototypic healthcare professional experiencing a differential mixture of multiple stressors. In addition to a significant workload, the stress associated with wanting to provide quality care (and not hurt anyone), and their own personal stressors, they are also the direct recipient of, or witness to, incivility from patients, family members, and other staff. A cumulative burden of stress worsens their negative affect (increasing irritation, sadness, anger, etc.) and depresses cognitive function. In turn, their diagnostic abilities, communication, and teamwork skills, as well as their problem-solving skills, all diminish [83,84,85,86]. Errors result, some of which will be substantial and cause harm and possibly death to a patient.

As errors occur, the clinician becomes a second victim, which in turn adds to their stress and further worsens cognitive processing and performance [87,88]. As this vicious cycle continues, it is not surprising that so many suffer from burnout, depression, and suicide. It is also known from the non-medical organizational behavior literature that workers who have been exposed to incivility, including from customers, bring their affect home with them and share it with their families, which is associated with decreased marital satisfaction [89]. Of course, as stress and dissatisfaction occur at home, the second victim brings those emotions back into work.

In conceptualizing the dynamics of this process, it is important to consider that emotions and behaviors are highly contagious [90]. Thus, it is predictable that stress, sadness, irritation, etc., will not just reside with one affected person. It will spread, just as incivility has been shown to be contagious [91,92].

What is particularly important is that training in health care emphasizes the ability to focus on the task at hand. One could have surmised that healthcare professionals would be more resistant to the effects of incivility given the importance of the patient’s health, especially in an emergency. That does not appear to be the case.

Ultimately, the human cost is huge—it is not difficult to imagine that, with higher functioning teams, more patients would have had a correct diagnosis made earlier and that correct therapy would have been initiated, resulting in fewer medical errors, recovery time, additional procedures, and disability and death.

The economic cost is substantial, too. Rawson and colleagues estimated that, at one hospital, the cost of disruptive and unprofessional behaviors exceeded USD 1 million annually. This included replacing staff as well as medication errors [93]. Simply adjusting that cost estimate (made in 2013) for the >6000 hospitals in the US would estimate ~USD 6 billion spent annually by hospitals, private insurers, and the government.

Another way to estimate the economic impact lies in the understanding that communication and teamwork issues are involved in over 70% of medical errors. Even if only 10% of medical errors were preventable by kindness and civility, and with a conservative estimate that the annual direct cost of medical errors is USD 30 billion, the direct cost of incivility is USD 3 billion.

What is not included in these cost estimates are malpractice settlements and costs to payers/employers for disability, time away from work, etc. For example, it is known that disruptive physician behaviors are tied to malpractice settlements [59,94]. For the annual indirect cost of harm in the US, in 2013, it was estimated at USD 1 trillion (OECD report). Even approaching these estimates in a conservative manner still illustrates that the economic consequences of incivility in health care are large for all stakeholders: patients, hospitals, staff, insurance companies (health and malpractice), as well as employers.

## 8. Stakeholder Involvement in the Problem

As detailed, there is an excessive and avoidable human and financial cost of disruptive behaviors. There are two enormous levers to drive change. Every stakeholder in healthcare should have a vested interest in changing this situation. To allow it to be perpetuated is a lose–lose scenario in one of the most critical workplaces that are encountered by nearly everyone.

The most important, of course, is the human cost of incivility. Patients want quality care that allows them and their loved ones to lead the highest quality of life possible. For nurses, physicians, and many others, the vast majority entered the profession to provide quality care using applied science. Committing a significant error has an enormous consequence for them, their sense of purpose and emotional energy to persevere [95]. It accelerates burnout and depreciates what they, in general, want to accomplish.

Yet, for some, a financial impetus is also needed. Hospital leaders need to recognize what disruptive behaviors could cost them in denied payments, staff issues and burnout, and patient retention issues through loss of reputation. Healthcare insurers need to consider extended hospital stays and added procedures and treatments, medication errors requiring emergency room visits and admissions, and much more. In addition, the problem of delayed (or mis-) diagnoses incurs additional testing, physician consultation, and perhaps complications because of delays.

For patients, as they need to cover deductibles, the costs of excess testing and medical errors will be shared with them. If they are disabled, even temporarily, that will also incur additional expenses. Finally, employers who pay premiums on behalf of their employees have a vested interest in keeping patients as healthy as possible (and at work) as well as keeping premiums down.

Malpractice insurers also need to consider the ramifications of promoting quality interpersonal relationships to diminish the likelihood of errors and injury. Annual malpractice paid claims are approximately USD 4–5 billion per year.

Overall, if we can decrease the number of errors by a conservative 10% and/or affect the severity of the error and the injury by decreasing disruptive behaviors, would that be considered beneficial to warrant finding ways to promote kindness?

## 9. Positive Emotions, Clinical Performance, and Cognitive Function

Beyond the impact of negative affect on decreasing performance, there is evidence that positive affect increases cognitive function and performance, i.e., there is potential that, beyond decreasing incivility, the error rate might drop even further by shifting staff the workplace’s affect to a net positive. Oppel and colleagues studied the relationship between a civil healthcare workplace and civility towards patients and their experience [96]. They found that there was a strong association between civility amongst staff and civility towards patients. They also observed a direct effect of civility climate on overall hospital rating, intent to recommend, and willingness to return.

Estrada and colleagues studied the effect of increasing positive affect on clinical performance [97]. Forty-four internists were randomized into one of three groups: control, positive affect induction, and humanism in medicine statements. They were to evaluate a case presented to them with the primary outcome variable as to how rapidly the physician arrived at the correct diagnosis versus anchoring on the wrong diagnosis. Compared to the control group, those provided the small bag of candy (which they did not eat) arrived at the correct diagnosis significantly faster.

In more general cognitive function studies, positive affect improves attention, creativity, and decision-making analogously to how negative emotions cause these to contract [98,99,100]. An observation of particular relevance to teamwork is the willingness to help others. Positive emotions such as eudaimonic joy (versus hedonism) increase helpfulness and kindness [99]. When combined with greater attention, creativity, and problem-solving, it is possible to envision higher-functioning teams, and as team performance rises, work-related satisfaction grows.

Related studies of anesthesia and critical care residents also support the improvement in cognitive function by lowering the stress response. Using relaxation techniques taught weeks before simulated surgeries, trainees performed significantly better during the simulation than those who were not taught these techniques [101,102,103]. Those familiar with the practices also had better recall of events of the simulation [103].

Stress is also less impactful when team members can rely on one another. This latter aspect was observed by Viotti and colleagues, who noted that “humanity of care” showed a positive and significant association with patient satisfaction. When the humanity of care was low, the impact of waiting time on patient experience was negative. When the humanity of care was medium or high, there was less impact of prolonged waiting time on patient experience [104].

## 10. Kindness Is Not Just about Being Nice

Thus, kindness is not just about being nice. It has very practical implications for interpersonal relationships, upon which many different outcomes depend. For patients, this includes satisfaction, trust, and engagement in the providers and their organization. For providers, it affects their own satisfaction as well as their cognitive function and hence decision-making, creative problem-solving, and ability to show compassion and caring. It will affect burnout and turnover. All of this has financial implications on multiple levels.

**Figure 2:**
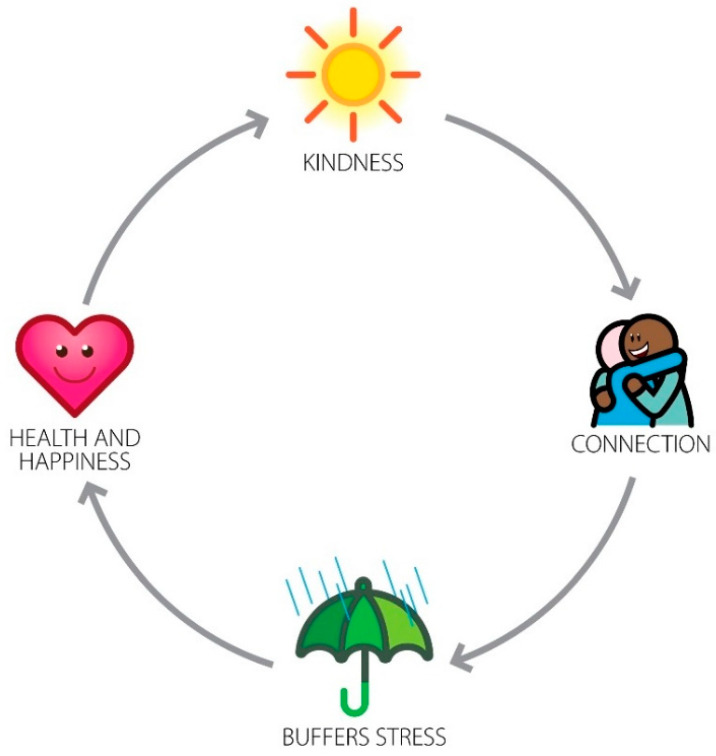
Kindness creates connection, which in turn, buffers stress and promotes resilience. As stress is attenuated, health and happiness become more manifest, which, in turn, creates more kindness. This is a virtuous cycle. Not shown in the figure is that simply seeing kindness, either in-person or in media, can trigger this cycle.

A simple(r) way to view this process is that kindness creates a positive interpersonal connection, which is well-known to buffer stress and promote resilience [105]. As the effects of stressors are lessened, better mental and physical health ensues. Happiness is a natural outcome of all of this, and happier people are kinder, completing a virtuous cycle [106]. Thus, the promotion of kindness has manifold implications for quality of life. This cycle is depicted in Figure 2.

Some may raise reservations about promoting kindness for a variety of reasons. One might be the misconception that the intrinsic nature of human beings is selfishness and that kindness is a distant priority or one that is externally imposed by a moral code or religion. Although all living beings have a drive for self-preservation (and selfishness), it is not the exclusive drive. The willingness to help others is present in many species and has been referenced by Darwin in his second book, *The Descent of Man*. That is, although self-interest is necessary for a single organism to survive, other-interest or preservation and the willingness to sacrifice for others, is necessary for the species to survive [107,108]. Nature has instilled that quality in all of us and is discussed extensively elsewhere [109]. We should look at human behavior as a modulation of these two drives and create the circumstances to allow for a greater manifestation of kindness and caring.

Another objection might be that medicine is about the focused application of science to help patients. That is how it is taught in graduate health care education. Invoking an entity as “soft” as kindness is antithetical to a data-driven, unemotional “just-the-facts,” and entirely rational approach to health care. It must be emphasized that fact-driven care is absolutely necessary. However, as discussed above, having just the facts is not enough. *Emotions clearly impact clinical decision-making*—the accession and interpretation of facts—in multiple ways. In addition to affecting cognitive function, they also affect the quality of the interaction between the patient and provider or healthcare team members who elicits and interprets those facts [110]. Facts alone, therefore, are insufficient for good care [77,86]. Given that health care is really about communication and trust, which are highly dependent on a connection, it is inescapable that kindness and caring are indispensable parts of health care.

Finally, kindness, in general, is relatively amorphous; it has many manifestations. Moreover, there may be less familiarity with the science surrounding it, including immunity, inflammation, vagal nerve activation, mortality, etc. These are detailed elsewhere [109] as well as described by Trzeciak and Mazzarelli [111]. One only needs to look at the literature on loneliness, volunteerism, and Blue Zones to know how positive interpersonal connection, a major outcome of kindness, is critical to health.

## 11. Promoting Kindness and Connection in the Health Care Workplace

Thus, there is a substantial warrant to foster kindness in the healthcare workplace actively. Many specific efforts have been developed to address this complex situation. They can be divided into three major categories, including: 1. Addressing the systemic issues that induce stress and cause burnout, depression, and suicide; 2. Supporting personal issues for staff regarding stress and the accompanying burnout; 3. Focusing on elements of the patient experience to enhance them.

Shanafelt and Noseworthy wrote an insightful overview of what healthcare leaders should consider for reducing stress and burnout for staff [112]. Specific initiatives have been shown to have promise, such as medical scribes to reduce administrative burdens for clinicians, among others [39,113,114], as well as broad recommendations [114].

Although specific initiatives can be quite impactful and foster kindness and lower stress in specific ways, prior to undertaking them, leaders should assess the culture first. Maslow recognized many years ago that beyond the achievement of basic needs (food, income, etc.), humans seek meaning, purpose, and belonging. That is, how do they matter? How do they have value and respect? As these are central to the human experience in promoting life satisfaction, then every aspect of work needs to be considered through this lens. Shanafelt and Noseworthy placed meaning at the center of the path to greater joy and engagement for staff [112].

Belonging, or feeling connected at work, is critical to performance and productivity and, ultimately, meaning. A Gallup poll of the general workplace has consistently shown the significance of having a “best friend at work” [115]. Those that had a best friend produced higher quality work, had greater well-being, and were seven times more likely to be engaged in their work [116]. What the best friend concept suggests is that people are connected to someone(s) else at work that cares about them. Imagine teams that are experiencing recurrent disruptive behaviors. On an individual contributor basis, their performance falls. As each affects the other, it is entirely anticipatable that their collective performance also falls. Conversely, teams that have positive interpersonal relationships and are in psychologically safe environments can flourish. When the team flourishes, everyone, especially the patient, benefits.

One way to create a more connected culture is kindness (prosocial behavior). There are recommendations that often start with leaders creating standards for expected behavior [117] and modeling behavior, including compliments, acknowledging others’ work publicly (and thanking them), practicing gratitude and appreciation, etc. What underpins making this easier to foster is the understanding that everyone needs to be respected [117]. They need to be seen and listened to, and treated honestly, including telling (and hearing) the truth even when it is uncomfortable. Psychological safety is critical.

On a day-to-day basis, those relationships and a sense of safety can be challenging to maintain as new events and stressors occur, and communication may be delayed. An entirely different way to complement the efforts above is to promote kindness and connection through kindness (prosocial) media. Studied in academic settings for many years, kindness media (such as a clip from Oprah) works rapidly (within 2 min) to “elevate” or uplift viewers. People experience a transcendent sense of being connected to others [118,119]. These studies have also shown that the decrease in the self–other gap is associated with the willingness to contribute to a charity supportive of members of another race [120] or express attitudes of greater acceptance of others.

A specific, novel portfolio of kindness media has been studied in different healthcare settings. It is intended for both patients and staff. The foundations of this portfolio are crowd-sourced images of kindness and caring from many cultures and countries that have been incorporated into short-form videos along with other types of media covering related concepts (gratitude, empathy, forgiveness) as well as quotes, etc. When streamed into the waiting room of a pediatric healthcare setting, this media rapidly uplifted both parents and staff and increased happiness, gratitude, and feeling calm. It increased generosity as measured by a donation to a needy family [121]. In other studies and settings, kindness media decreases irritation, stress, anxiety, and sadness and increases feeling connected to others as well as positively affects how hospital nursing and medical staff treat one another (unpublished results).

The media works well in all of these studies simply because we are “wired” to respond to it, similarly to how images of food or drink induce hunger or thirst. As media can stir this innate drive to want to connect with others and care for them, no training or significant time is needed in a stressed and time-constrained environment. As media work rapidly and can be streamed almost anywhere, it creates the possibility to help people reconnect quickly and on a regular basis with their own humanity and the humanity of others.

One aspect of the use of media in health care is that it is meant for both patients and staff. That is, as both patients and staff have stress, the intent is to create a better emotional and spiritual environment for both. Having both of these major stakeholder groups involved enhances the likelihood that a more positive and productive interaction will occur. Each party benefits from the improved countenance of the other and predicts a higher quality, more satisfying interaction. Eventually, it is anticipated this will translate to better clinical outcomes for patients (greater patient engagement, fewer errors) and happier and more satisfied staff. The next steps for testing the effects of these media include determining the impact on patient satisfaction, patient-provider communication, provider satisfaction, and teamwork.

## 12. Conclusions

By understanding that how we treat one another has critical and practical ramifications for both the mental and physical health of patients and staff, as well as for the cost of care, there is a unique and potential opportunity to improve health care for the better. At a minimum, the diminution of incivility would likely result in improved communication, satisfaction, and outcomes for both patients and staff. The relative simplicity of kindness and connection, which is derived from ancient wisdom rather than new technology, can allow for further enhancement of the quality of care with comparatively little cost.

## Data Availability

Not applicable.
